# The relationship between action, social and multisensory spaces

**DOI:** 10.1038/s41598-023-27514-6

**Published:** 2023-01-05

**Authors:** Laurie Geers, Yann Coello

**Affiliations:** CNRS, UMR 9193 - SCALab - Sciences Cognitives et Sciences Affectives, Université de Lille, Domaine Universitaire du Pont de Bois, BP 60149, 59653 Villeneuve d’Ascq Cedex, Lille, France

**Keywords:** Neuroscience, Cognitive neuroscience, Sensory processing, Social behaviour, Social neuroscience, Somatosensory system

## Abstract

Several spaces around the body have been described, contributing to interactions with objects (peripersonal) or people (interpersonal and personal). The sensorimotor and multisensory properties of action peripersonal space are assumed to be involved in the regulation of social personal and interpersonal spaces, but experimental evidence is tenuous. Hence, the present study investigated the relationship between multisensory integration and action and social spaces. Participants indicated when an approaching social or non-social stimulus was reachable by hand (reachable space), at a comfortable distance to interact with (interpersonal space), or at a distance beginning to cause discomfort (personal space). They also responded to a tactile stimulation delivered on the trunk during the approach of the visual stimulus (multisensory integration space). Results showed that participants were most comfortable with stimuli outside reachable space, and felt uncomfortable with stimuli well inside it. Furthermore, reachable, personal and interpersonal spaces were all positively correlated. Multisensory integration space extended beyond all other spaces and correlated only with personal space when facing a social stimulus. Considered together, these data confirm that action peripersonal space contributes to the regulation of social spaces and that multisensory integration is not specifically constrained by the spaces underlying motor action and social interactions.

## Introduction

The space immediately surrounding the body is of foremost importance for any living being as it is the space in which physical interactions with the environment take place. During the last decades, countless studies in cognitive neurosciences have fortified the idea that the representation of space is functional, i.e., the space offering information on the possibilities of acting on objects must be processed differently by the brain than the space offering information on the mere presence of objects with no possibility of acting on them. This view has led to the distinction between *peripersonal space* (PPS, i.e., within reach) and *extrapersonal space* (i.e., beyond reach)^1,2^, which would be underpinned by different neural networks^3,4^. The concept of PPS originates from single-unit electrophysiological studies in monkeys showing that a number of neurons within the ventral premotor cortex, the parietal cortex and the putamen responded more to objects presented in the near reachable space than objects presented in the far unreachable space^2,5,6^. Thus, PPS has been conceived as an interface between the body and the environment, contributing to the organisation of object-directed motor actions, either in terms of approach when facing incentive objects or in terms of avoidance when facing threatening objects^7,8^. In line with this, neuroimaging studies revealed that the mere observation of an object located in PPS triggered activation in the sensorimotor brain areas, including the reach-related area of the superior parieto-occipital cortex, and the premotor and motor cortical areas^9–12^. As a consequence, the transient disruption of the left motor cortex using transcranial magnetic stimulation has been shown to produce an alteration of the perception of objects located in PPS^13^. Likewise, corticospinal activity^14^ and *μ* rhythm desynchronization^15,16^ increased in the presence of objects in the near (vs. far) space, similar to what has been observed during the preparation and execution of objects-directed motor actions^17,18^. Moreover, modifying the actual reaching-by-hand capabilities (*e.g.,* through tool-use or limb immobilisation), or biasing the spatial consequences of object-directed actions, entailed a congruent increase or decrease of the PPS^19–22^. Altogether, these results suggest that PPS is an action space, enabling access to motor-related information similar to those implied in the planning and execution of voluntary motor actions^23,24^.

As revealed by monkey electrophysiological studies, most PPS neurons are multisensory in that they respond to stimuli in two or three different sensory modalities, with overlapping receptive fields anchored onto the same body region^2,5,6^. In addition, neural and behavioural investigations have consistently shown that stimuli in one sensory modality enhance the processing of stimuli in another modality, especially when those stimuli are perceived as potentially interacting with our body^8^. Importantly, some of these neurons are particularly responsive to a tactile stimulation delivered in co-occurrence with an approaching visual stimulus, provided the two stimuli fall in the neuron’s receptive fields^2^. This multisensory integration is of particular relevance for interactions with the environment, which require the position of external stimuli to be combined with information about different body segments^23^, as reflected by higher-order activations of somatosensory and associative areas^25^. Such multisensory integration has also been observed in humans, activating a frontoparietal network^26–28^, in relation to PPS^29^. However, the main line of evidence in humans supporting multisensory integration in relation to PPS comes from behavioural studies showing that the proximity of a visual/auditory stimulus from a certain body region fastens the detection of tactile stimulation on that body region, and the maximal distance at which such facilitation is observed (as compared to a unisensory control condition) is usually used as a proxy of the PPS extent^30–33^. The scientific consensus is that the integration of visual/auditory and tactile information would provide an interface between perception and action allowing appropriate (re)actions towards (either threatening or incentive) objects to be generated. The relevance of multisensory integration to action preparation and execution is indeed supported by the studies on the effect of permanent or temporary damage to the monkey's cortex showing a direct relationship between the PPS multisensory network and the accuracy of motor responses^34–37^. Furthermore, electric stimulation of the PPS multisensory neurons in the monkey premotor and intraparietal cortex elicited a pattern of movements that is compatible with defensive arm movements^38^, while the PPS multisensory neurons in the parietal and precuneus cortex have been shown to discharge during arm reaching movements towards the part of space corresponding to their visual receptive field^2^. PPS represents thus a multisensory and sensorimotor interface mediating the physical interactions between the body and the environment^39^.

Hence, if PPS consists in a multisensory interface dedicated to physical interactions with the environment, the reachable and multisensory integration spaces are expected to overlap. However, the wealth of studies on behavioural multisensory facilitation in humans has highlighted a high degree of lability of the multisensory integration space, depending notably on the body region targeted by the tactile stimulation^33^. Indeed, when considering similar experimental conditions (i.e., the detection of a tactile stimulus in the presence of a looming auditory stimulus), the extent of the multisensory integration space tended to be shorter when the tactile stimulus was delivered on the hand (around 40 cm), than on the face (around 50 cm) or trunk (around 55 cm). Moreover, it is worth noting that the range of distances leading to multisensory integration varied considerably across studies, even when using the same experimental conditions (from 20 to 66 cm for the hand, from 17 to 86 cm for the head; from 25 to 80 for the trunk^30–33,40–53^. Hence, multisensory integration does not seem to systematically overlap with the motor action space. In support of this claim, Zanini and colleagues^54^ found that the space corresponding to hand-centred visuotactile integration was shorter than the space reachable with the hand, and moved with the hand, while reachable space was insensitive to hand position. They concluded that multisensory and reachable spaces are distinct spatial representations. However, it is worth underlying that the observed dissociation might also arise from the different frames of reference involved in the two tasks. It is indeed known that object-directed action involves a trunk-centred or eye-centred frame of reference, but not a hand-centred frame of reference^55–58^. By contrast, multisensory integration was tested using a hand-centred frame of reference that requires, for motor action, to refer to a more global representation of the body constituting the egocentre^33,39,59,60^. Accordingly, the “trunk-centred” reachable-by-hand space was not expected to coincide exactly with the “hand-centred” multisensory integration space. In line with this claim, Serino and colleagues^33^ considered that “hand- and face-centred [multisensory] PPS are referenced to the trunk-centred [multisensory] PPS, which [is] a more extended representation of the space surrounding the body”. Hence, multisensory integration might be compatible with the representation of the space that is reachable with the hand when referring to the same frame of reference, i.e., a trunk-centred frame of reference, which has never been truly tested.

Another important aspect of the body-environment interactions concerns the nature of the stimulus under consideration. Studies in social psychology have focused on interactions with conspecifics instead of physical objects, and have typically divided the space around the body in a series of bubbles that serve to maintain proper spacing between individuals. The smallest bubble is the *personal space* (PS)*,* which is defined as the space in which social intrusion is felt to be threatening or uncomfortable^61^. It is assumed to serve as a margin of safety around the body and is typically assessed with discomfort distance judgments requiring the participants to judge at which distance a confederate makes them uncomfortable^62–65^. A second and larger bubble is the *interpersonal space* (IPS), which is defined as the space one maintains between oneself and others during social interactions^66^. It is typically assessed with comfort distance judgments requiring the participant to place a confederate at the most comfortable distance to interact with^67,68^. Not only do these social spaces refer to the space surrounding the body as PPS, but also share common characteristics with PPS. For instance, PPS is modulated by social factors such as the proximity of confederates and the relation that is held with them^47,69–71^. Furthermore, both PPS and social spaces shrink or enlarge depending on the emotional valence of the facing stimulus^62,69,72^. They are also both influenced by individual characteristics such as anxiety^64^. These observations probably explain why several researchers in the last decades have taken a closer look at the relationship between PPS and social spaces. Until now, studies have mainly focused on the link between PPS and PS. For instance, Iachini and colleagues^63,73^ reported that both spaces have a similar size (around 50 cm) and are similarly affected by the nature, age, and gender of the stimulus. They reported that both PPS and PS reduce with humans as compared to robots and cylinders, with females as compared to males, and with children as compared to adults. It has therefore been proposed that PPS, and more particularly its sensorimotor and multisensory properties, serves as a spatial anchor to calibrate social distances^23,74,75^. In support of this claim, Quesque and colleagues^65^ found that extending arm's length representation through tool-use increased PPS with a concomitant effect on PS. Social spaces seem thus rooted in the same sensorimotor representation as PPS^23^. However, the above-mentioned studies have mainly focused on the relative impact of different factors on the PPS and social spaces, which provides little information about their relationship. Moreover, these studies did not include a measure of IPS and thus failed to provide a comprehensive picture of the extent of the different social spaces and their relationship to PPS. Finally, the involvement of multisensory integration in social spaces has not yet been studied in depth.

In this context, the present study investigated the relationship between the different action and social spaces anchored on the body and multisensory integration. Participants had to indicate when an approaching neutral visual stimulus (human, robot or lamp) was reachable with the arm (indexing reachable space, RS), at the most comfortable distance to interact with (indexing interpersonal space, IPS), or started to generate discomfort due to too much proximity (indexing personal space, PS). We also included a visuotactile integration task that required participants to respond as fast as possible to a tactile stimulation delivered on the trunk at various times of the approach of the visual stimulus, while ignoring the latter (indexing multisensory integration space, MIS). We expected RS to overlap and correlate with MIS, as being two representative measures of the trunk-centred PPS. Also, along with the idea that the regulation of social distances is based on PPS representation^23,74,75^, we expected RS and MIS to correlate with PS and IPS, although IPS should be larger and PS should be smaller than RS and MIS^63,65,73^. Finally, all spaces should be similarly impacted by the nature of the stimulus, with the lamp and robot being kept at a larger distance compared to the human^73^.

## Materials and methods

### Participants

Fifty-three participants from the Université of Lille participated in this study, but one participant was excluded because they missed 20% of the tactile stimulations in the multisensory integration task, and two others were excluded because they showed no multisensory facilitation effect, making it impossible to compute its location in space. The final sample was thus composed of 50 participants (12 males, mean [*M*] age ± standard deviation [*SD*] = 22.6 ± 4.0). A sample size analysis performed in G*Power indicated that at least 41 participants were required to detect a small effect (Cohen’s *f* = 0.15) with a high power criterion (0.9) in a 4 × 3 repeated-measure ANOVA. All participants were right-handed and had a normal or corrected-to-normal vision. They all gave written informed consent prior to the experiment. The study was performed in accordance with the ethical standards of the Declaration of Helsinki and was approved by the Research Ethics Board of the University of Lille (CESC Lille, Ref. 2021–515-S95).

### Apparatus and stimuli

**Figure 1:**
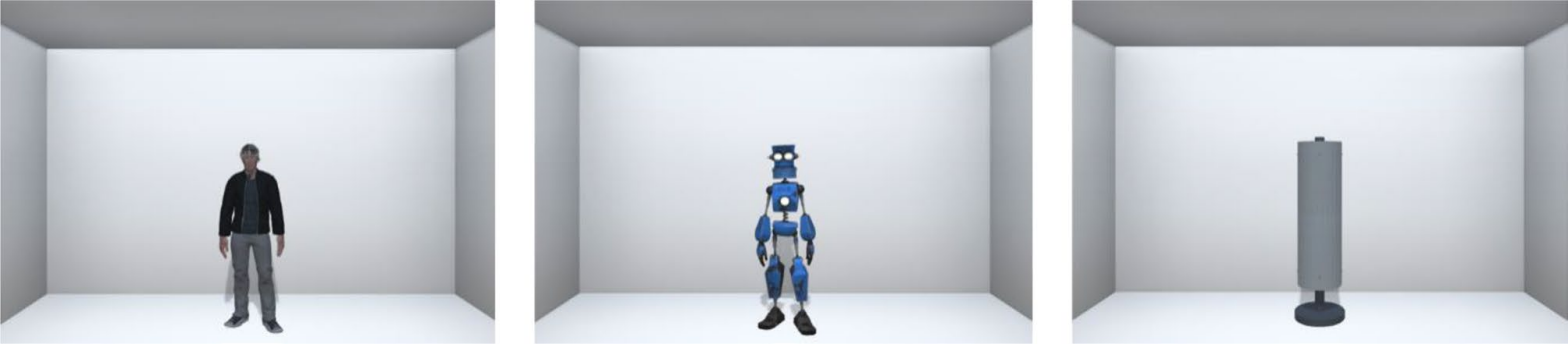
The virtual environment and stimuli used in the four tasks: a neutral human adult male, an anthropomorphic robot and a cylindrical lamp appearing at 300 cm in front of the participant in an undecorated and unequipped room.

The virtual stimuli were presented through an HTC Vive Pro head-mounted display in a virtual room measuring 6 × 5 × 3 m, and consisting of a white floor, a grey ceiling and grey walls. The stimuli consisted of a human male avatar aged about 30 years, an anthropomorphic robot and a cylindrical lamp. The man and robot looked straight ahead and showed a neutral facial expression (Fig. 1). The height of the stimuli was calibrated so that the eye level of the human and robot were aligned with the eye level of the participant. All stimuli had the same height and width. We verified that the visual stimuli were perceived as neutral by requiring the participants to rate the emotional valence of each stimulus on the Self-Assessment Manikin (SAM) scale, a 9-points graphic Likert scale ranging from 1 (extremely negative) to 9 (extremely positive)^76^. One sample *t*-test to 5 (i.e., neutral emotional valence) indicated that the human, *t*(49) = 0.47, *p* = 0.643, robot, *t*(49) = 1.85, *p* = 0.071, and lamp, *t*(49) = -1.24, *p* = 0.220, were similarly judged as neutral.

### Tasks & procedure

Participants were standing while holding a response button in their right hand, and wearing the head-mounted display. A vibrotactile stimulator (DRV2605 Haptic Driver, Texas Instruments) was fixed to their sternum with an elastic band. They performed the four following tasks in a counterbalanced order:

#### Reachability distance judgment

Participants were required to press the response button as soon as they judged being able to reach the approaching visual stimulus, without actually performing any reaching movement. Each trial started with the appearance of a visual stimulus at 300 cm in front of the participant for a duration of 500 ms, which then approached the participant at a velocity of 0.75 m/sec. Whenever the participant pressed the response button, the visual stimulus stopped moving and remained still for 1000 ms before disappearing. The next trial started at a random delay between 800 and 850 ms following the disappearance of the previous stimulus. The task consisted of 18 trials (3 stimuli × 6 repetitions), lasted about 2 min, and was used to assess RS.

#### Comfort distance judgment

The same procedure as in the reachability distance judgment task was used, except that participants were required to press the response button as soon as the visual stimulus was judged at the most comfortable distance to interact with it. This task was used to assess IPS.

#### Discomfort distance judgment

*T*he same procedure as in the reachability distance judgment and comfort distance judgment tasks was used, except that participants were required to press the response button whenever the visual stimulus was at a distance that made them feel uncomfortable. This task was used to assess PS.

#### Multisensory integration task

Participants were required to respond as quickly as possible to a tactile stimulation (60 ms, 3.6 V, 250 Hz) delivered well above the detection threshold on their sternum while ignoring the visual stimulus facing them. The task included 4 types of trials: bimodal visuotactile, unimodal tactile, bimodal catch and unimodal catch trials. In all types of trials, the visual stimulus appeared at 300 cm in front of the participants for 500 ms. In the bimodal visuotactile trials, the stimulus moved towards the participants at a velocity of 0.75 m/sec. A tactile stimulation was delivered at one of the 8 following delays: 1333, 2000, 2267, 2533, 2800, 3067, 3333 or 3600 ms after the setting in motion of the visual stimulus. This means that the visual stimulus was respectively at 200, 150, 130, 110, 90, 70, 50 and 30 cm from the participant at the time the tactile stimulation occurred. Hence, the longer the delay, the closer the stimulus from the participants. In the unimodal tactile trials, the tactile stimulation was provided after 1333, 2800 or 3600 ms, but the visual stimulus remained still. These trials served as a baseline and allowed us to investigate the facilitation effects induced by the spatial proximity of the visual stimulus in the bimodal trials while controlling that these effects were not merely due to the expectancy of tactile stimulation or attention varying with temporal delay. In the bimodal catch trials, the visual stimulus moved toward the participant until being at a distance of 20 cm, but no tactile stimulation was delivered. In the unimodal catch trials, the visual stimulus remained still, but no tactile stimulation was delivered. These catch trials were included to avoid automatic motor responses and make sure that the participants were attentive to the task all along the experiment. Whenever the participant pressed the response button, the visual stimulus stopped moving and remained still for 1000 ms before disappearing. The next trial started at a random delay between 800 and 850 ms following the disappearance of the previous stimulus. The whole task consisted of 414 trials, including 240 visuotactile bimodal (3 stimuli × 8 delays × 10 repetitions), 90 unimodal (3 stimuli × 3 delays × 10 repetitions), 42 bimodal catch (3 stimuli × 14 repetitions) and 42 unimodal catch (3 stimuli × 14 repetitions) presented in random order. The trials were divided into 6 blocks of about 6 min intermingled with 5-min breaks. This task was used to assess MIS.

### Data analyses

The data were analysed using *R* (version 4.1.0) and *R Studio* software (version 1.3.1093). We first verified that our multisensory integration task succeeded in showing the typical effects of the tactile stimulation delay on reaction times (RT) in each of the three stimuli used (see Supplemental Materials for procedure and results).

#### Extent of the different spaces

To determine the individual extent of RS, PS and IPS, we averaged for each participant and each stimulus the distance of the visual stimulus at the time of the response in the reachability judgement task, and in the discomfort and comfort distance judgement tasks, respectively. The extent of MIS was determined by identifying the farthest distance at which the bimodal trials induced facilitation effects as compared to the unimodal trials in the visuotactile integration task (see Supplemental Materials for detailed procedure). We then compared the different spaces in terms of their average extent and their sensitivity to the nature of the visuals stimulus by entering the computed extents in a repeated-measures ANOVA with the Space (RS, MIS, IPS, PS) and type of Stimulus (human, robot, lamp) as within-subject variables. Since the extent of MIS was an ordinal variable and the extent of the different spaces, as well as the residuals of the model, did not follow a normal distribution, we used an Aligned Rank Transform (ART) for nonparametric factorial ANOVAs as described by Wobbrock and colleagues^77^. We planned to conduct pairwise comparisons on the significant effects, but also on the effect of the Stimulus on each task, to investigate whether we replicate the observation of expanded PPS and PS in the presence of a virtual human as compared to a virtual robot and a lamp^63,73^ when using stimuli controlled for their (neutral) emotional valence. The paired comparisons were performed using the ART^77^ or ART-C^78^ alignment procedure, as appropriate to the requested contrast, and with Bonferroni correction.

#### Relationship between the different spaces

We then further investigated the relationship between the different spaces with pairwise correlation analyses. We computed the correlation coefficients for each stimulus separately. In particular, we computed Pearson *r* coefficients, except when correlation included MIS, in which case we computed the Spearman *r* correlation coefficient for ordinal variables.

#### Bayesian analyses

We also conducted the corresponding Bayesian analyses in JASP (with default values) in order to quantify the evidence in favour of an effect (H1) compared to an absence of effect. These analyses provided Bayes Factors (*BF*_10_) varying between 0 and ∞, where values below 1 provide increasing evidence in favour of the null hypothesis and values above 1 provide increasing evidence for the alternative hypothesis (H1/H0)^79^. A BF above 3 is typically considered sufficient evidence for the alternative hypothesis, while a BF below 1/3 is typically considered sufficient evidence for the null hypothesis^80^.

## Results

### Extent of the different spaces

**Figure 2:**
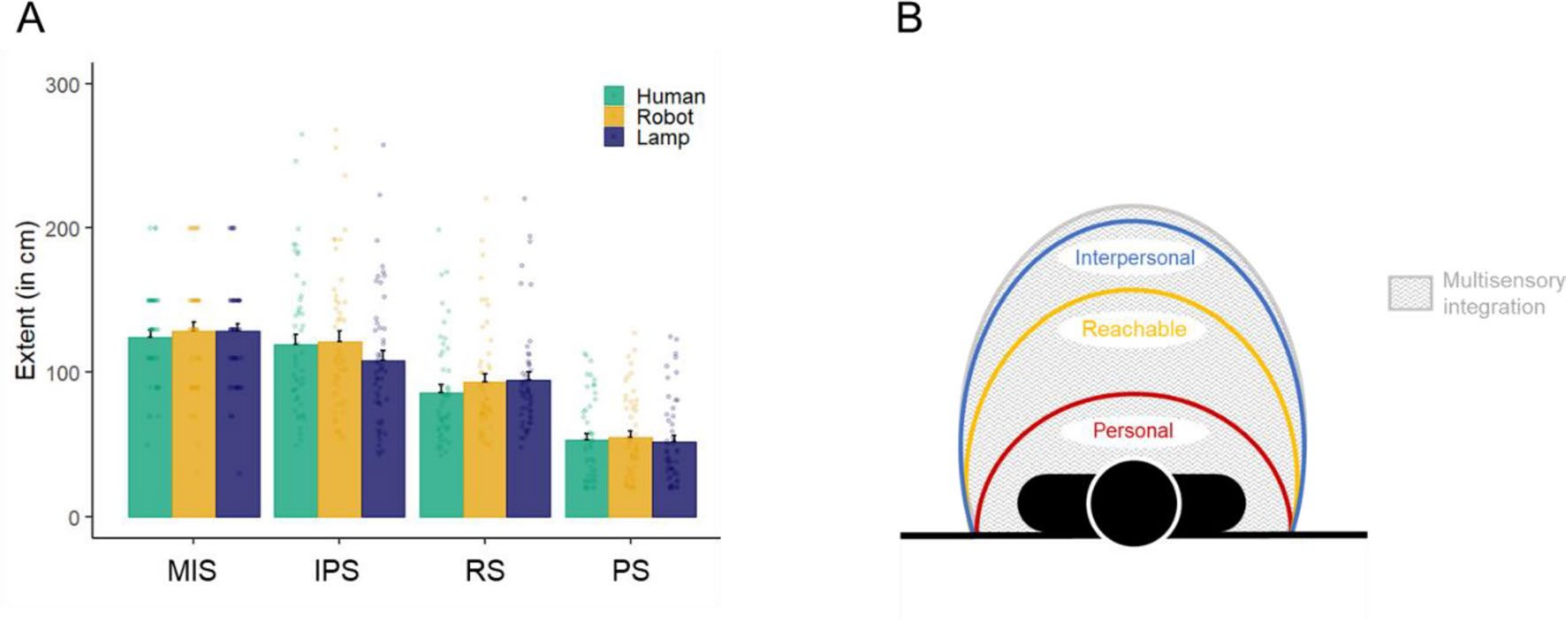
(**A**) The extent of the different spaces (MIS, IPS, RS, PS) expressed in centimetres as a function of the stimulus (human, robot, lamp). The bars represent the average extent (error bars represent the SE), while the dots represent the individual performances. (**B**) Schematic representation of the organisation of the different spaces.

The ANOVA comparing the extent of the different spaces and the sensitivity to the different visual stimuli showed a significant effect of Space, *F*(3,539) = 181.31, *p* < 0.001, *η*_*p*_^2^ = 0.502, *BF*_10_ = 6.94^+^^66^. The average extent ± standard error (SE) was 127.40 ± 2.98 cm for MIS, 116.35 ± 4.05 cm for IPS, 91.36 ± 3.08 cm for RS and 53.47 ± 2.37 cm for PS. Post hoc pairwise comparisons showed that all spaces were significantly different from each other (all *p*-values < 0.001; Fig. 2A). There was no significant effect of the Stimulus, *F*(2,539) = 0.62, *p* = 0.536, *η*_*p*_^2^ = 0.002, *BF*_10_ = 0.04, or Space by Stimulus interaction, *F*(6,539) = 0.73, *p* = 0.626, *η*_*p*_^2^ = 0.008, *BF*_10_ = 0.02. The planned comparisons, however, showed a significant effect of the Stimulus on RS, *F*(2, 98) = 13.84, *p* < 0.001, *η*_*p*_^2^ = 0.220, *BF*_10_ = 2018.02, with participants judging the human as reachable at shorter distances (M ± SE = 86.24 ± 5.04 cm) than the robot (93.16 ± 5.55 cm), *t*(98) = –3.43, *p* = 0.002, *BF*_10_ = 3835.21, and the lamp (94.66 ± 5.41 cm), *t*(98) = -5.17, *p* < 0.001, *BF*_10_ = 355.93, while RS for the robot and lamp did not significantly differ from each other, *t*(98) = -1.74, *p* = 0.195, *BF*_10_ = 0.205. The effect of Stimulus was also significant for IPS, *F*(2, 98) = 6.88, *p* = 0.002, *η*_*p*_^2^ = 0.123, *BF*_10_ = 36.62. Post-hoc pairwise comparisons further indicated that participants preferred to place the lamp at shorter distances (108.43 ± 6.65 cm) than the robot (121.34 ± 7.34 cm), *t*(98) = -3.42, *p* = 0.003, *BF*_10_ = 14.78, and the human (119.27 ± 6.98 cm), *t*(98) = –2.95, *p* = 0.011, *BF*_10_ = 3.02, while the preferred distance for the human and robot did not significantly differ from each other, *t*(98) = -0.47, *p* = 0.884, *BF*_10_ = 0.271. By contrast, the effect of the Stimulus was marginal (or null, according to Bayesian analyses) on PS, *F*(2, 98) = 3.01, *p* = 0.054, *η*_*p*_^2^ = 0.057, *BF*_10_ = 0.290, with only the lamp being tolerated closer than the robot, *t*(98) = -2.45, *p* = 0.047, *BF*_10_ = 0.56. Finally, the effect of Stimulus on multisensory space was not significant, *F*(2, 98) = 0.28, *p* = 0.752, *η*_*p*_^2^ = 0.005, *BF*_10_ = 0.089.

### Relationship between the different spaces

**Figure 3:**
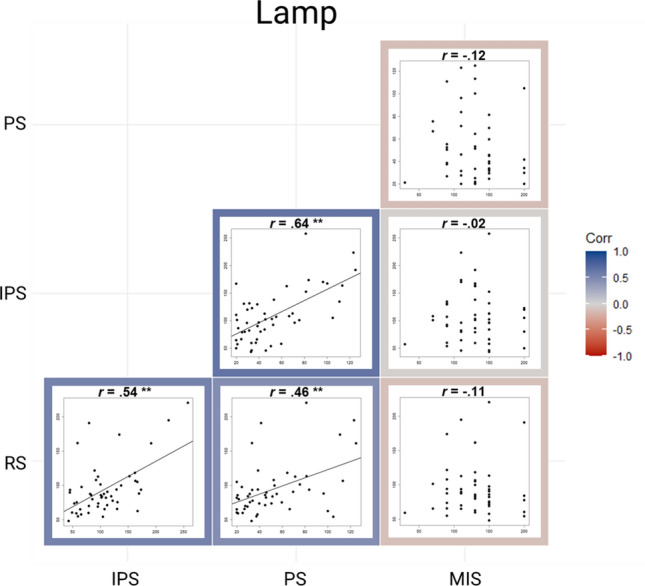
Correlation matrix plot showing the relation between RS, IPS, PS and MIS when facing the virtual lamp. The *r* refers to the Spearman coefficient when the correlation includes MIS and to the Pearson coefficient when it does not. **p-values < .001, * *p*-values < .05.

**Figure 4:**
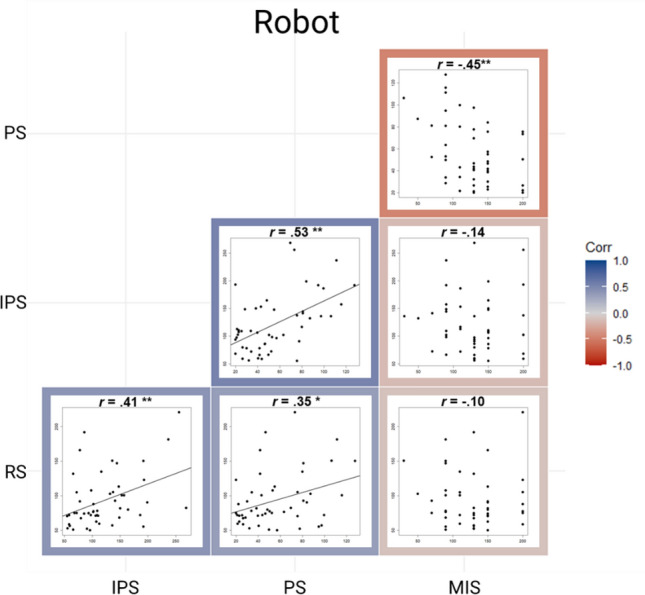
Correlation matrix plot showing the relation between RS, IPS, PS and MIS when facing the virtual robot. The *r* refers to the Spearman coefficient when the correlation includes MIS and to the Pearson coefficient when it does not. **p-values < .001, * *p*-values < .05.

**Figure 5:**
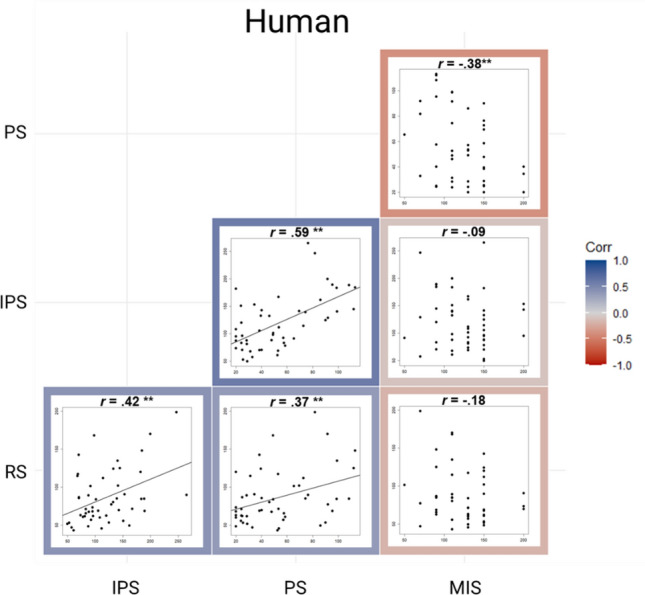
Correlation matrix plot showing the relation between the RS, IPS, PS and MIS when facing the virtual human. The *r* refers to the Spearman coefficient when the correlation includes MIS and to the Pearson coefficient when it does not. **p-values < .001, *p-values < .05.

Regarding the lamp, a significant positive correlation was found between RS and IPS, *r* = 0.54, *p* < 0.001, *BF*_10_ = 594.58, between RS and PS, *r* = 0.46, *p* < 0.001, *BF* = 42.33, as well as between IPS and PS, *r* = 0.64, *p* < 0.001, *BF*_10_ = 31,768.77. The correlation between RS and MIS was not significant, *r* = -0.11, *p* = 0.443, *BF*_10_ = 0.28, so as the other correlations including MIS (all *p*-values > 0.407, all *BF*_10_-values < 0.27; Fig. 3). Regarding the robot, we also found a significant positive correlation between RS and IPS, *r* = 0.41, *p* = 0.003, *BF*_10_ = 11.23, RS and PS, *r* = 0.35, *p* = 0.014, *BF*_10_ = 3.38, as well as between IPS and PS, *r* = 0.53, *p* < 0.001, *BF*_10_ = 335.11. In addition, there was a significant negative correlation between PS and MIS, *r* = -0.45, *p* < 0.001, *BF*_10_ = 63.84. No other correlation was significant (all *p-*values > 0.938, all *BF*_10_ < 0.31), including the correlation between RS and MIS, *r* = -0.10, *p* = 0.499, *BF*_10_ = 0.23 (Fig. 4). Regarding the human, we found the same significant correlations as in the robot: a positive correlation between RS and IPS, *r* = 0.42, *p* = 0.003, *BF*_10_ = 13.92, between RS and PS, *r* = 0.37, *p* = 0.007, *BF*_10_ = 5.40, PS and IPS, *r* = 0.59, *p* < 0.001, *BF*_10_ = 4169.65, as well as a negative relation between PS and MIS, *r* = -0.38, *p* = 0.006, *BF*_10_ = 8.94. There was no other significant correlation (*p*-values > 0.210, *BF*_10_-values < 0.439; Fig. 5).

## Discussion

The aim of the present study was to assess the relationship between the reachable and multisensory spaces, two representative measures of the trunk-centred PPS, and how the latter related to the social spaces. To do so, we required participants to indicate when an approaching neutral visual stimulus (human, robot or lamp) was reachable with the arm (RS), at the most comfortable distance to interact with (IPS), or too close so that it generated a feeling of discomfort (PS). We also included a visuotactile integration task (MIS) that required participants to respond as fast as possible to tactile stimulation delivered on the trunk at various times of the approach of the visual stimulus. Based on the idea that PPS is an action space characterised by sensorimotor and multisensory properties, we expected the extent of RS and MIS not only to overlap but also to correlate. Moreover, along with the idea that PPS contributes to the regulation of the social spaces, we expected all spaces to correlate and to be similarly impacted by the nature of the stimulus, although PS should have the smallest extent and IPS the largest one. The results showed that the extent and relationship of the reachable and social spaces were in line with our hypotheses, supporting the idea that RS serves as a spatial reference to define the social spaces. Contrary to expectations, MIS exceeded RS and did not correlate with it, suggesting that multisensory processes do not specifically characterize RS.

In particular, the analyses of the extent of the different spaces showed that PS (58 cm) was smaller than RS (95 cm), which was, in turn, smaller than IPS (121 cm, Fig. 2B). This indicates that, as expected, both objects and humanoids were preferentially placed outside RS to interact with, and generated discomfort when present well inside it^81,82^. Note that it is common for RS to exceed arm length^83^ (here 73.2 ± 5.6 cm, corresponding for RS to an overestimation of 29%), in particular in virtual environments^84^. Unlike what was expected, reachable and social spaces were not all similarly impacted by the nature of the stimulus. As already shown^63,73^, RS was significantly shorter in the presence of a virtual human than in the presence of a lamp or robot (7.7 cm in the present study). This confirms that PPS representation expanded with virtual objects and reduced with virtual humans. Conversely, the extent of IPS was not different between the robot and human, and significantly shorter with the lamp (11.9 cm in the present study). This might reflect the fact that interactions with objects require touching them and thus be at shorter distances than people for which interactions might be primarily conceived as a verbal exchange, especially when the situation involves a stranger^66^. The lack of difference between the robot and human stimulus might suggest that the anthropomorphic aspect of the robot used in the present study was sufficient to consider social interaction with it. In this case, it is expected that human-like stimuli with the same (neutral) emotional valence should be positioned at the same interpersonal distance^62,74,81^. A complementary interpretation could be that the human stimulus used in the present study was a male who was shown to trigger larger IPS than a female human stimulus^63,73^. Further experiments would be required to disentangle these different interpretations. Note that the differential impact of the stimulus’ nature on reachable and social spaces, although in contradiction to our initial hypotheses, is not necessarily in conflict with the idea of a relationship between those spaces, which is further discussed below.

Despite these differences in extent and sensitivity to the nature of the stimulus, RS, IPS and PS were positively correlated with each other, as expected. This means that the participants with a larger RS were also those who had a larger IPS and PS, and conversely, whichever the stimulus presented. These data confirm previous studies highlighting that the regulation of PS depends in some respect on the representation of PPS^65^, although the outcome of the present study extends the contribution of PPS also to IPS. The observed pattern of results, therefore, provides an additional argument for the involvement of PPS in the calibration of social spaces^63,65,73,74^, and corroborates brain imaging studies showing that the frontoparietal network involved in the representation of PPS also supports social interactions^85,86^. Overall, these findings comfort the idea that action and social spaces are related but more specifically that the sensorimotor properties of PPS serve as a spatial reference to specify the appropriate social distances, as suggested by the homeostatic theory of social interactions^74^. According to this theory, the appropriate inter-individual distance corresponds to PPS plus an extra margin of safety, that adapts according to the valence or level of threat endowed on conspecifics. This theory, therefore, accounts for the observation that IPS correlates with RS but has a larger extent. In its original form, the theory did not take into account PS and assumed that PPS is a protective buffer zone whose intrusion produces discomfort^81,82^ and triggers defensive behaviour^87^. As discussed above, the present study rather underlines that discomfort is experienced when stimuli are well inside RS. PS is therefore a better candidate if we consider the priority space dedicated to the protection of the body, although it seems calibrated from PPS representation minus a tolerance margin that would allow for PPS intrusion, at least to some extent, which is often required during interactions both with objects and living beings. This theory also allows taking into account the differential effect of the stimulus nature on PS, IPS and RS, by allowing some aspects to have specific effects on the margin of safety of the IPS or PS.

The striking result of the present study is however the observation that trunk-centred multisensory integration extended much further away than RS (+ 36.04 cm), which is in contradiction with our initial hypothesis. MIS extent is furthermore much larger in the present study than what was previously observed with auditory stimuli when also using a trunk-centred frame of reference (i.e., around 55 cm, from 25 to 80)^33^. One potential explanation could be that multisensory integration extends more when facing meaningful visual stimuli. A careful inspection of previous studies supports this hypothesis: hand-centred and face-centred multisensory integration were found to be both more extended when facing virtual human characters (up to 127 and 150 cm, respectively)^88,89^ than when facing looming pink noise (up to 66 and 75 cm, respectively)^43,50^. However, even when centred on the same trunk-centred frame of reference as the reachability task, MIS did not correspond to RS. This indicates that multisensory integration is not specifically related to the motor action space. These findings contrast with the single-cell recording studies in monkeys showing that the receptive fields of the multisensory neurons are within RS^2^. However, one may hypothesise that the sensory facilitation reported in the behavioural studies and the neural mechanisms highlighted in the single-cell studies do not refer to the exact same multisensory integration process^90^. While the link between the two has been strongly advocated^30^, it is apparent that the behavioural multisensory facilitation effect in humans is more flexible than what was reported in single-cell studies. As evidence, multisensory facilitation in behavioural studies has been found to be altered by the valence or meaning of the visual/auditory stimulus^69^, individual traits such as anxiety/phobia^46^, interoceptive traits^41^, bodily changes such as pregnancy^42^ or limb immobilisation^91^, and even lockdown experience^88^. Moreover, a number of studies indicated that the visual/auditory stimulus does not have to target the same body part as the tactile stimulation to trigger multisensory facilitation^90^. This might be because the behavioural effects evidenced arose not only from the multisensory brain areas but also from their interaction with other brain areas such as those involved in body representation^92^ and object-directed action control^7^. Another aspect of the behavioural studies on humans is that they implied a task-dependent motor response, while monkeys were generally studied in a passive condition. Thus, despite their pioneering role, single-cell studies might represent only a small window onto the network underpinning multisensory integration in the context of goal-directed motor action. This may explain the lack of correlation that we found between MIS and RS, corroborated by the Bayesian analysis, albeit single-cell studies revealed a link between multisensory integration and arm RS^2,5^. It might be argued that the lack of correlation arose from a requirement to respond to different sensory signals, i.e., the visual stimulus in the reachability task and the tactile stimulation in the multisensory integration task. However, the extent of both spaces reflects the influence of the spatial proximity of the visual stimulus on the response. Moreover, if visuotactile integration processes truly characterise RS, we should have observed this correlation despite any procedural difference. From a behavioural perspective, it seems thus that RS refers to a different spatial representation than MIS despite being tested with a typical looming task and using the same spatial frame of reference. PPS, as an action space, must thus be viewed as a sensorimotor interface anchored on the body that involves, but does not depend on, multisensory integration.

Importantly, MIS also extended further away than PS (+ 73.93 cm) and IPS (+ 11.05 cm). This implies that multisensory processes, usually related to the action space, extend also to the social space. This is not that surprising since the need to combine several sensory cues is not restricted to interactions with objects but also applies to social stimuli. For instance, emotions are expressed through facial expressions but also voice such that visual and auditory cues integration is an essential part of emotion reading and more globally of social interactions^93^. Moreover, multisensory integration is assumed to allow the preparation of the body for action, either for the purpose of defensive or approaching behaviour^7,8,73,74^. Physical contact with people, though less frequent than with objects, is also experienced on a daily base: we shake hands, hug, are tapped on the shoulder to get our attention, or brush against each other in crowded environments, with some of these contacts, for instance when concerning people with bad intentions, being at risk for the body. The functional advantage provided by multisensory integration is thus also relevant for social interactions to anticipate possible contact with others and programme appropriate actions and responses — for example, to avoid harmful contact or shake hands properly with our interlocutor. A consequence of this approach is that multisensory integration must be viewed as a process at hand during interactions with either objects or individuals, which is not specific to the nature of the present stimulus or the type of interaction envisaged, and which thus seems not constitutive of the spaces underlying object-directed actions and social interactions. Moreover, the negative correlation found between MIS and PS for humanoid stimuli (human and robot), could suggest that multisensory integration serves mostly a defensive purpose^8^. In particular, people characterised by a larger MIS were also characterised by a shorter PS, which may reveal an adaptive link between anticipation of physical contact with social stimuli and acceptance of the proximity of these stimuli. In other words, individuals that integrate visual information with tactile information earlier (i.e., at further distances) might be better prepared to react to those visual stimuli and thus tolerate them closer. It should be noted that this correlation was not observed for the lamp, which might arise from the fact the lamp is, in principle, static and thus less threatening. However, those interpretations are speculative at this stage and require further investigation.

In conclusion, this first study comparing PPS (RS and MIS) and the social spaces (PS and IPS) showed that only the action PPS was related to the social spaces. This finding confirms previous studies reporting that RS and PS are related^63,65,73,81^, but extends this relationship to IPS. This further underlines the particular role of the sensorimotor aspects of PPS in the regulation of the social spaces, providing new evidence in support of the homeostatic theory of social interactions^74^. Multisensory integration was not restricted to action PPS and social spaces, as it extended beyond all these spaces. This indicates that multisensory integration is involved in interactions with objects and people, in relation to the anticipatory aspects of these interactive behaviours, but does not specifically determine the representation of both action PPS and social spaces. The specific role of multisensory integration in the different interactions with the environment, therefore, remains to be further clarified.

## Supplementary Information

Supplementary Information.

## Data Availability

All data analysed in this study have been made publicly available on Open Science Framework (OSF) via the following link: https://osf.io/xp9r8/?view_only=ed8daecc5dfa43b8b1a024abdb37bb2f.
