# Supplementary material for: The relationship between action, social and multisensory spaces
Source: Sci Rep. 2023 Jan 5;13:202. doi: 10.1038/s41598-023-27514-6 (PMC9814785; doi:10.1038/s41598-023-27514-6)
Supplement: Supplementary file 1 — Supplementary Information. [file 41598_2023_27514_MOESM1_ESM.pdf]

The relationship between action, social and multisensory spaces: Supplemental Materials

Geers, Laurie[a] & Coello, Yann[a]

[a]CNRS, UMR 9193 - SCALab - Sciences Cognitives et Sciences Affectives, Université de Lille, Lille, France

Multisensory integration task

Check-up analyses and results

Before computing the extent of the multisensory integration, we verified that our paradigm succeeded in showing the typical effects of the tactile stimulation delay on reaction times (RTs)[1,2,3,4,5,6,7] in each of the three stimuli used. To do so, we first excluded trials with responses prior to tactile stimulation (*i.e.,* false alarm; 0.66 % of the data), with no response (*i.e.,* misses; 0.99 % of the data) and with reaction times (RT) lower or higher than 2 SD from the participant's mean (4.18 % of the data). Catch trials only served to avoid automatic motor responses and were excluded from further analyses. We then checked whether the delay of the tactile stimulation affected the unimodal and/or bimodal trials by including the remaining RTs in a repeated-measure ANOVA including the Delays that were common to both type of trials (3; 1333, 2533 vs. 3600 ms) and the type of Trial (2; unimodal vs. bimodal) as within-subjects variables.

The ANOVA on the RT when facing the lamp showed a main effect of Delay, $F(2, 98) = 110.8$, $p < .001$, $\eta_p^2 = .69$, indicating that the greater the delay of the tactile stimulation the shorter the RTs. Post hoc paired t-tests further indicated that, compared to when tactile stimulation was delivered at 1333 ms (331 ± 5 ms), the RTs were faster at 2800ms (296 ± 6 ms), $t(98) = 12.22$, $p < .001$, and 3600 ms (293 ± 6 ms), $t(98) = 13.47$, $p < .001$, while there was no difference between RTs to stimulation delivered at 2800 and 3600 ms, $t(98) = 1.25$, $p =$

.645. There was also a significant effect of the type of Trial, $F(1, 49) = 357.6$, $p < .001$, $\eta_p^2 = .88$, indicating that RTs were faster for the bimodal (279 ± 4 ms) than for the unimodal (334 ± 4 ms) trials. Finally, there was a significant Delay by Trial interaction, $F(2, 98) = 49.7$, $p < .001$, $\eta_p^2 = .50$, showing, after decomposition, that the effect of the delay was greater for the bimodal, $F(2, 98) = 133.5$, $p < .001$, $\eta_p^2 = .73$, than for the unimodal trials, $F(2, 98) = 4.2$, $p = .017$, $\eta_p^2 = .08$. Note, however, that the effect of Delay was significant for the unimodal trials, with RTs at 1333 being slower than at 2800 ms, $t(98) = 2.70$, $p = .025$, indicating that there is an effect of the temporal delay that is independent of the spatial proximity of the stimulus.

The ANOVA on the RT when facing the robot showed a main effect of Delay, $F(2, 98) = 87.45$, $p < .001$, $\eta_p^2 = .64$, indicating that the greater the delay of the tactile stimulation the shorter the RTs. Post hoc paired *t*-tests further indicated that all delays were significantly different from each other ($p < .006$). There was also a significant effect of the type of Trial, $F(1, 49) = 216.7$, $p < .001$, $\eta_p^2 = .82$, indicating that RTs were faster for the bimodal (274 ± 4 ms) than for the unimodal (333 ± 5 ms) trials. Finally, there was a significant Delay by Trial interaction, $F(2, 98) = 36.5$, $p < .001$, $\eta_p^2 = .43$, showing, after decomposition, that the effect of the delay was greater for the bimodal, $F(2, 98) = 160.2$, $p < .001$, $\eta_p^2 = .77$, than for the unimodal trials, $F(2, 98) = 6.7$, $p = .002$, $\eta_p^2 = .12$. Note, however, that the effect of delay was significant for the unimodal trials, with RTs at 1333 being slower than at 2800 ms, $t(98) = 3.09$, $p = .008$, and 3600 ms, $t(98) = 3.26$, $p = .004$, indicating that there is an effect of the temporal delay that is independent of the spatial proximity of the stimulus.

The ANOVA on the RT when facing the human showed a main effect of Delay, $F(2, 98) = 116.6$, $p < .001$, $\eta_p^2 = .70$, indicating that the greater the delay of the tactile stimulation the shorter the RTs. Post hoc paired *t*-tests further indicated that, compared to when tactile stimulation was delivered at 1333 ms (330 ± 5 ms), the RTs were faster at 2800ms (295 ± 5 ms), $t(98) = 12.12$, $p < .001$, and 3600 ms (290 ± 6 ms), $t(98) = 14.11$, $p < .001$, while there was no difference between RTs to stimulation delivered at 2800 and 3600 ms, $t(98) = 1.99$, $p =$

.147. There was also a significant effect of the type of Trial, $F(1, 49) = 202.8$, $p < .001$, $\eta_p^2 = .81$, indicating that RTs were faster for the bimodal (281 ± 4 ms) than for the unimodal (329 ± 4 ms) trials. Finally, there was a significant Delay by Trial interaction, $F(2, 98) = 57.1$, $p < .001$, $\eta_p^2 = .54$, showing, after decomposition, that the effect of the delay was greater for the bimodal, $F(2, 98) = 172.6$, $p < .001$, $\eta_p^2 = .78$, than for the unimodal trials, $F(2, 98) = 10.8$, $p < .001$, $\eta_p^2 = .18$. Note, however, that the effect of delay was significant for the unimodal trials, with RTs at 1333 being slower than at 2800 ms, $t(98) = 4.48$, $p < .001$, and 3600 ms, $t(98) = 3.29$, $p = .004$, indicating that there is an effect of the temporal delay that is independent of the spatial proximity of the stimulus.

Our results thus replicated previous observations of an effect of tactile stimulation on RTs that is more important in bimodal than unimodal trials. This confirms the need to account for the part of the effect of temporal delay that is independent from spatial proximity by subtracting the fastest mean of the unimodal trials from the RTs of the bimodal trials[5]. The observation that the effect of temporal delay on the unimodal trials is not identical for each stimulus further underlines the need to perform this correction for each stimulus individually (see below).

Extent computation

The extent of multisensory integration was determined by identifying the farthest distance at which the bimodal trials induced facilitation effects as compared to the unimodal trials. To do so, we averaged, for each participant and stimulus, the RT in the unimodal trials over the tactile stimulation delay, and identified the smallest mean, which was then subtracted from the bimodal RT of that stimulus. Negative and positive values thus indicate bimodal facilitation and interference, respectively, compared to the fastest unimodal delay (*i.e.,* baseline [0]), which allows controlling for the effect of temporal delay independent of the spatial proximity of the stimulus. Supplemental Figure 1 illustrates the observed facilitation

effects (both at the individual and group level) averaged over all stimuli as a function of its distance from the participant at the time of the stimulation. Finally, we compared the multisensory facilitation effect against 0 (*i.e.,* unimodal baseline) using paired *t*-tests in each participant, character and delay. The size of the multisensory space was defined as the shortest delay (and thus farthest distance) at which the t-test was significant.

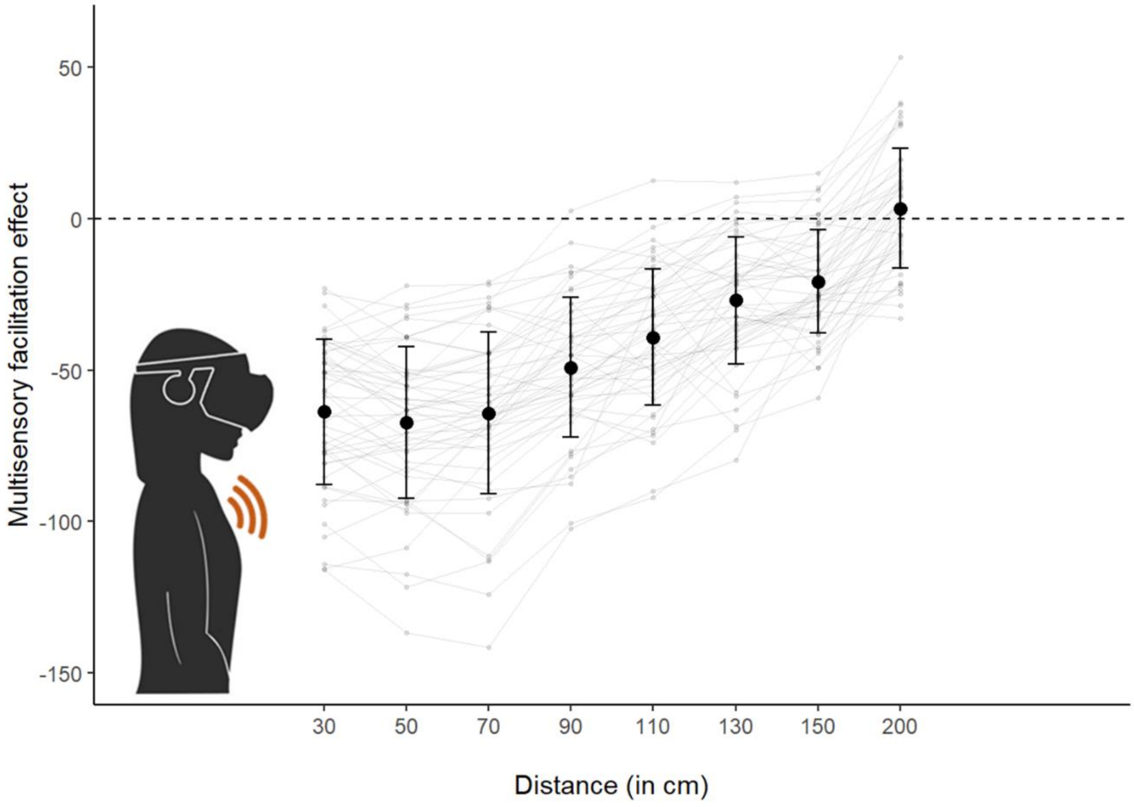

Supplemental Figure 1. Multisensory facilitation effect as a function of the distance between the visual stimulus and the participant at the time of the tactile stimulation. The small gray dots represent the effects at the individual level. These individual effects are used to compute the extent of multisensory integration (*i.e.,* farthest distance at which the facilitation effect is significantly different from the unimodal baseline level represented by the dashed line). The large black dots represent the effect averaged over participants (error bars represent the SD).

References

1. Canzoneri, E. *et al.* Tool-use reshapes the boundaries of body and peripersonal space representations. *Exp Brain Res* **228**, 25–42 (2013).
2. de Haan, A. M., Smit, M., Van der Stigchel, S. & Dijkerman, H. C. Approaching threat modulates visuotactile interactions in peripersonal space. *Exp Brain Res* **234**, 1875–1884 (2016).
3. Kandula, M., Van der Stoep, N., Hofman, D. & Dijkerman, H. C. On the contribution of overt tactile expectations to visuo-tactile interactions within the peripersonal space. *Exp Brain Res* **235**, 2511–2522 (2017).
4. Pellencin, E., Paladino, M. P., Herbelin, B. & Serino, A. Social perception of others shapes one's own multisensory peripersonal space. *Cortex* **104**, 163–179 (2018).
5. Serino, A. *et al.* Body part-centered and full body-centered peripersonal space representations. *Sci Rep* **5**, 18603 (2015).
6. Serino, S. *et al.* Sharpening of peripersonal space during the COVID-19 pandemic. *Current Biology* **31**, R889–R890 (2021).
7. Stone, K. D., Kandula, M., Keizer, A. & Dijkerman, H. C. Peripersonal space boundaries around the lower limbs. *Exp Brain Res* **236**, 161–173 (2018).
